# The Effect of Nanobubble Water Containing Cordyceps Extract and Withaferin A on Free Fatty Acid-Induced Lipid Accumulation in HepG2 Cells

**DOI:** 10.3390/nano13152265

**Published:** 2023-08-07

**Authors:** Hanlin Han, Yixin Sun, Weixu Zhang, Zhenya Zhang, Tian Yuan

**Affiliations:** Graduate School of Science and Technology, University of Tsukuba, 1-1-1 Tennodai, Tsukuba 305-8572, Ibaraki, Japan; s2130296@u.tsukuba.ac.jp (H.H.); s2126059@s.tsukuba.ac.jp (Y.S.); s2330336@u.tsukuba.ac.jp (W.Z.); zhang.zhenya.fu@u.tsukuba.ac.jp (Z.Z.)

**Keywords:** nanobubble water, non-alcoholic fatty liver disease, cordyceps extract, withaferin A, HepG2, lipid reduction

## Abstract

Cordyceps extract and withaferin A (Wi-A) are natural compounds that have therapeutic effects on non-alcoholic fatty liver disease (NAFLD). However, their efficacy is limited and a long treatment duration is usually required. To enhance their efficiency, the synergistic effects of nanobubble water (NBW) derived from nitrogen, hydrogen, and oxygen gases were investigated. Results showed that the physical properties of all three NBWs, including nanobubble density (10^8^ particles/mL) and zeta potential (below −22 mV), were stable during 48 h of storage. Hydrogen and nitrogen NBWs did not reduce, but instead promoted, free fatty acid-induced lipid accumulation in HepG2 cells. In contrast, oxygen NBW synergistically enhanced the effects of cordyceps extract and Wi-A. The lipid content decreased by 29% and 33% in the oxygen NBW + cordyceps extract and oxygen NBW + Wi-A groups, respectively, compared to reductions of 22% and 16% by aqueous extracts without NB. This study found that NBW may enhance the lipid-reducing effects of natural compounds, such as cordyceps extract and withaferin A, in hepatic cells. Further studies in animal experiments are needed to determine whether NBW has a potential application in NAFLD.

## 1. Introduction

Non-alcoholic fatty liver disease (NAFLD) includes a range of liver abnormalities, such as non-alcoholic fatty liver (NAFL) and non-alcoholic steatohepatitis (NASH), which can finally lead to cirrhosis and liver cancer [1]. Nowadays, NAFLD has become one of the most common diseases in young adults. According to a previous report, 24% of adults in the United States have NAFLD [2]. Despite the increased prevalence of NAFLD, effective approaches for the treatment of NAFLD that go beyond the advice to increase exercise and control diet [3] are still sought after. Although some compounds have been used to treat NAFLD, they have limited efficacy and/or are associated with certain side effects. Sanyal et al. used pioglitazone, vitamin E, and a placebo to treat NASH and found that pioglitazone and vitamin E led to a great improvement in patients. However, the study noted that the long-term use of vitamin E may lead to an increased risk of cardiovascular disease [4]. Pioglitazone also has the long-term side effect of weight gain [5]. In recent years, glucagon-like peptide-1 (GLP-1) analogs and sodium-glucose co-transporter type-2 (SGLT2) inhibitors have been approved for the treatment of NAFLD [6,7]. However, some studies have shown that GLP-1 analogs are accompanied by certain side effects, such as vomiting and gastrointestinal disorders [8]. SGLT2 inhibitors were also found to increase the risk of reproductive tract infections [6]. Microbial therapy using probiotics and prebiotics has also been trialed in the treatment of liver disease, but the mechanisms of action have not yet been elucidated [9,10]. In addition, the high cost of chemical drugs increases the healthcare burden and limits their application in therapy.

Considering the side effects of chemical drugs on the human body and the cost, researchers have turned their attention to natural compounds. Among the many natural compounds, cordyceps extracts and withaferin A (Wi-A) have been studied extensively owing to their various bioactivities. Li et al. found that cordyceps extract reduced lipid accumulation in the liver by activating the adenylate acylase/protein kinase A (PKA) signaling pathway and inhibiting the mammalian target of rapamycin (mTOR) signaling pathway [11]. Wi-A has been shown to inhibit lipid accumulation in HepG2 cells by modulating liver X receptor alpha (LXR-α) and farnesoid X receptor (FXR) [12]. Although these two compounds can reduce or inhibit lipid accumulation, their effects are limited. In fact, a high concentration of cordyceps extract and Wi-A was applied in these studies, which might be harmful to human body. In addition, the prolonged use of drugs can cause serious financial burden and resistance in the body [13]. However, long-term medication is the basis of the treatment of chronic diseases. Therefore, highly effective and nonharmful treatment is preferable in today’s fast-paced society, presenting the challenge of improving the efficacy of natural compounds in the treatment of NAFLD.

Various approaches have been tried to improve the efficacy of natural compounds. Among them, the synergistic effect of nanomaterials has drawn increasing attention. Nanomaterials have been extensively investigated because of their smaller size, surface activity, ability to enter cells through a variety of pathways, and modulable drug delivery properties by changing shapes [14]. In recent years, nanobubble water (NBW) has been extensively investigated as a novel nanomaterial. It has been shown that oxygen NBW could provide an oxygen-rich growth environment for aerobic microorganisms and accelerate the formation of biofilms [15]. In addition, NBW could also improve the production of probiotics by facilitating the transport of substances [16]. In the case of cancer treatment, it has been also reported that NBW, individually or in combination with other substances, could inhibit cancer cell proliferation by inducing apoptosis and promoting oxidative stress [17,18]. Owen et al. found that oxygen NBW could reduce the size of solid tumors by regulating the expression of hypoxia-inducible factor 1-alpha (HIF1α), vascular endothelial growth factor (VEGF), and other related proteins [19]. In contrast, some researchers have also found that oxygen NBW reduces the level of ROS in cells, thereby reducing the degree of oxidative stress [20].

Although NBW has been applied in a variety of fields, there is a knowledge gap in the treatment of NAFLD. Moreover, no study has been found to investigate the synergistic effect of NBW with other drugs on the treatment of NAFLD. Considering the unique physiological properties of NBW, it is necessary to investigate its therapeutic effect on NAFLD and its role in promoting the efficacy of natural compounds for the treatment of NAFLD.

In this study, the physical properties of three types of NBW (nitrogen, hydrogen, and oxygen) were first investigated. Then, the effect of NBWs and their synergistic effects with two natural compounds, cordyceps extract and Wi-A, on lipid reduction in NAFLD model cells were evaluated.

## 2. Materials and Methods

### 2.1. Cells and Reagents

HepG2 cells (American Type Culture Collection, Manassas, VA, USA) were used in this experiment to construct the NAFLD cell model. Wi-A (Tokiwa Phytochemicals Co., Ltd., Chiba, Japan) was dissolved using dimethyl sulfoxide (DMSO, Wako, Japan) and a 5 mM stock solution was prepared. Cordyceps extract (DKS Co., Ltd., Tokyo, Japan) was dissolved using ultrapure water (Direct-Q 3UV Water Purification System, Merck, Tokyo, Japan). After adding 1.36 g cordyceps extract in 10 mL of sterilized ultrapure water, the insoluble impurities were filtered out using a 0.22 µm filter membrane. The concentration of solubilized extract was determined by subtracting the weight of insoluble extract left on the filter membrane. Then, a 10 mg/mL stock solution was prepared using sterilized ultrapure water.

### 2.2. Preparation of NBW

NBW was produced using an ultra-fine nanobubble generator (HACK FB11, LIVINGENERGIES & CO., Shizuoka, Japan). First, 1.0 L of deionized water was circulated through the circulation generator, and three different gases (oxygen, nitrogen, and hydrogen) were introduced, respectively. Due to the negative pressure of the pump, the gases were sucked into the pump and mixed with water at a high speed to produce nanobubbles. The production time was 20 min [21], during which the outlet pressure was maintained at 0.25 ± 0.05 MPa to keep the water milky white. After that, the produced water was left on the table until the water became clarified, which meant that most of the microbubbles disappeared while the nanobubbles remained.

### 2.3. Determination of the Properties of NBW

The prepared NBWs were stored in 500 mL glass bottles for 48 h at 37 °C. During the storage, the particle size and density of nanobubbles were determined using a nanoparticle tracking analysis detector (NanoSight-LM10, Malvern, UK). The surface charge of nanobubbles in NBWs was measured using a zeta potential analyzer (NanoZS, Malvern, UK). The amount of dissolved oxygen (DO) in the NBW was measured using a DO meter (HQ40d, Hach Company, Loveland, CO, USA).

### 2.4. Preparation of Culture Medium Containing NBW

The 10 times concentrated (10×) Dulbecco’s Modified Eagle Medium (DMEM) was prepared using powdered DMEM (Gibco, Waltham, MA, USA) with high glucose content according to the product instructions. The fully dissolved medium was sterilized using a sterile disposable filter unit (Thermo Fisher Scientific, Suite, PA, USA). The solution was stored in a refrigerator at 4 °C. The prepared NBWs were sterilized by using 0.45 μm filter membranes [16]. Then, 10× DMEM was diluted to 1× DMEM using sterilized ultrapure water and NBW at different concentrations (20%, 40%, 60%, 80%, 90%).

### 2.5. Cell Viability Assay

HepG2 cells were cultured in high-glucose DMEM supplemented with 10% fetal bovine serum (FBS, Gibco, USA) and 1% penicillin–streptomycin–amphotericin B suspension (×100) (Wako, Japan) in an incubator (37 °C, 5% CO_2_). The cultured cells were inoculated in 96-well plates at 5000 cells per well and cultured overnight. Then, the cells were treated with 1 mM free fatty acids (FFAs) for 24 h to establish a fatty liver cell model. HepG2 cells with sufficient lipid accumulation were treated with three different types of NBW (20%, 40%, 60%, 80%, 90%), cordyceps extract (0.1, 0.2, 0.4, 0.8 mg/mL) and Wi-A (0.1, 0.2, 0.4, 0.8 µM) at different concentrations for 48 h, respectively. Subsequently, 3-(4,5-dimethyl-2-thiazolyl)-2,5-diphenyl-2-H-tetrazolium bromide (MTT) was added to the medium (5 mg/mL). After incubation for 4 h, the medium in each well was removed and 100 µL DMSO was added into each well. The absorbance of the solution was measured at 450 nm.

### 2.6. Nile Red Assay for Determination of Intracellular Lipid

The non-cytotoxic concentrations of NBW and natural compounds confirmed by cell viability assay have been further adopted to treat the FFA-exposed HepG2 cells.

After treatment, cells in the plates were washed 3 times with phosphate-buffered saline (PBS, Wako, Japan). Then, the cells were fixed with 4% paraformaldehyde phosphate buffer solution (PFA) for 20 min at room temperature (25 °C). Subsequently, PFA was aspirated and the residual PFA was removed by rinsing with PBS 3 times. The intracellular lipids were stained with 1 µg/mL Nile Red solution at 25 °C for 5 min. The image of the stained cells was obtained by a fluorescence microscope (Leica DM-IRB, Leica, Tokyo, Japan), and the area of intracellular lipids stained in red was quantified using ImageJ (http://imagej.org/, 10 February 2023).

### 2.7. Data Analysis

All the data are expressed as the mean ± standard deviation (SD) calculated from data of three independent experiments, and the experimental data showed a normal distribution. In addition, statistical analysis was conducted using unpaired *t*-test. * *p* < 0.05, ** *p* < 0.01, and *** *p* < 0.001 were considered as statistically significant.

## 3. Results

### 3.1. Physical Properties of NBW

**Figure 1:**
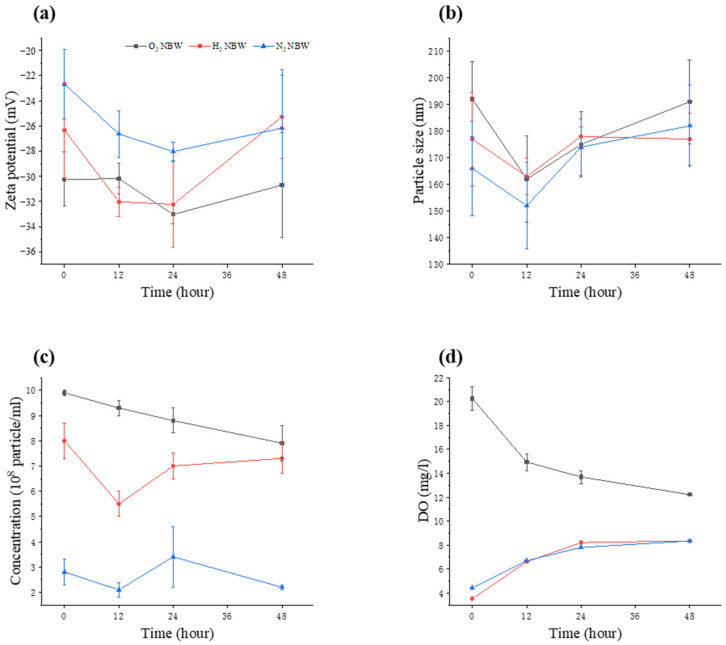
Changes in physical properties of NBW. (**a**) Zeta potential; (**b**) particle size; (**c**) nanobubble concentration; (**d**) dissolved oxygen (DO).

Since the effect of NBW on lipid reduction in FFA-exposed HepG2 cells was tested for 48 h in this study, the changes in various physical properties of NBW were measured during a 48-h storage period (Figure 1). It could be seen that the zeta potential of NBWs did not change significantly during the 48 h (Figure 1a). Although hydrogen NBW and nitrogen NBW led to fluctuations in zeta potential values, the statistical analysis revealed that there were no significant differences, and the zeta potential values at 48 h were essentially the same as those at 0 h. Oxygen NBW showed the smallest fluctuation during 48 h, maintaining at around −30 mV. The particle size of the nanobubbles in the three NBWs remained below 200 nm and did not change significantly during the experiment (Figure 1b).

The alteration of the nanobubble concentration of the oxygen NBW tended to differ from the hydrogen and nitrogen NBWs (Figure 1c). The initial content of oxygen nanobubbles was the highest among the three NBWs and it gradually decreased from 9.9 × 10^8^ particles/mL to 7.9 × 10^8^ particles/mL during the storage. The hydrogen and nitrogen nanobubble concentrations remained essentially unchanged during 48 h. Moreover, the number of nanobubbles at 48 h was essentially the same as that at 0 h. Although the concentrations of nanobubbles were different and changed in the three types of NBW over 48 h, the order of magnitude did not change significantly and remained at 10^8^ particles/mL. Figure 1d shows the changes in dissolved oxygen (DO) for the three types of NBW over 48 h. The oxygen NBW had the highest DO value. Although the DO of the oxygen NBW decreased, it remained at a higher level during the 48 h than those of hydrogen and nitrogen NBWs. The DO values of hydrogen and nitrogen NBWs and the changes during 48 h were almost the same, gradually increasing over 24 h and then stabilizing at 8 mg/L.

### 3.2. Cytotoxicity Evaluation

**Figure 2:**
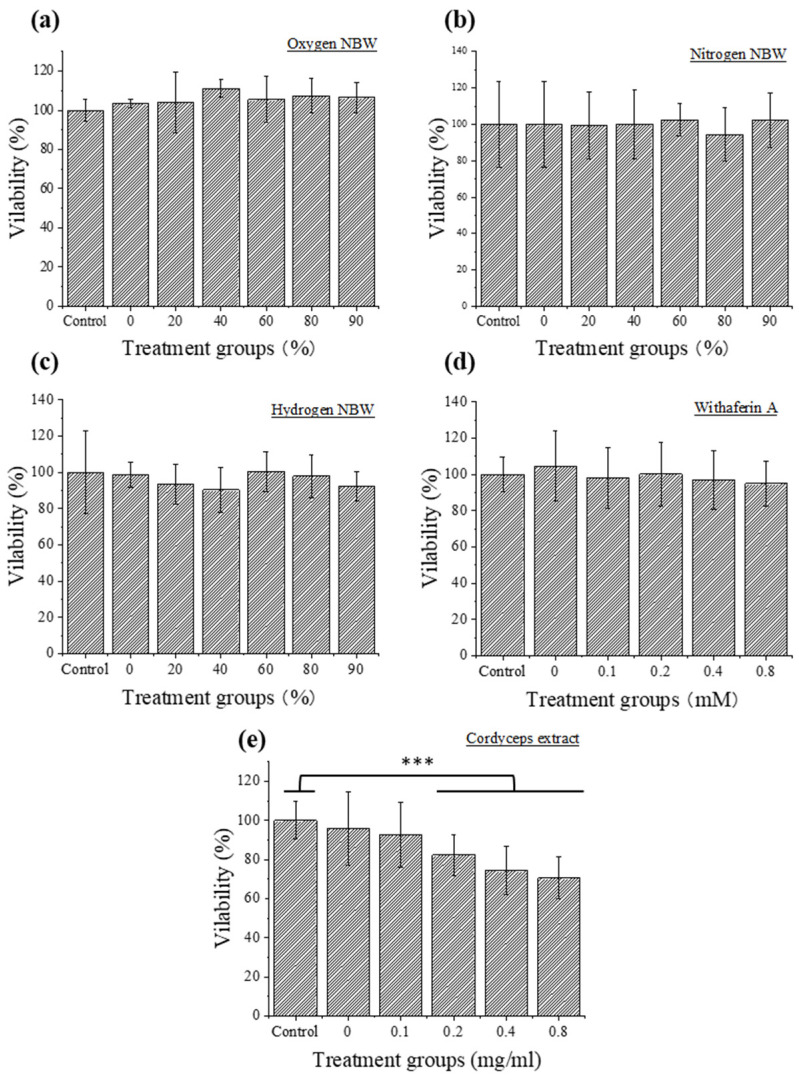
Cytotoxicity evaluation of nitrogen (**a**), hydrogen (**b**), and oxygen (**c**) NBWs (0–90%), withaferin A (Wi-A) (0–0.8 µM, (**d**)) and cordyceps extract (0–0.8 mg/mL, (**e**)) on FFA-exposed HepG2 cells. Control denotes HepG2 cells with no FFA treatment. Significant differences in cell viability at each condition were compared to that of control. *** indicates significant difference at *p* < 0.001.

Figure 2 shows the results of the cytotoxicity evaluation of NBWs and natural compounds on the FFA-exposed HepG2 cells. The results indicated that none of the three NBWs at concentrations of 0–90% was toxic to FFA-exposed HepG2 cells. To maximize the synergistic effects of NBWs with the natural compounds on lipid reduction, 90% NBW was selected for subsequent experiments. Similar to the cytotoxicity results of NBWs, different concentrations of Wi-A were not toxic to FFA-exposed HepG2 cells. Thus, 0.8 µM Wi-A was adopted in the following experiments. However, cordyceps extract at concentrations of above 0.2 mg/mL significantly reduced cell viability. Thus, 0.1 mg/mL of cordyceps extract was selected for the subsequent experiments.

### 3.3. Synergistic Effect of Nitrogen NBW with Natural Compounds

**Figure 3:**
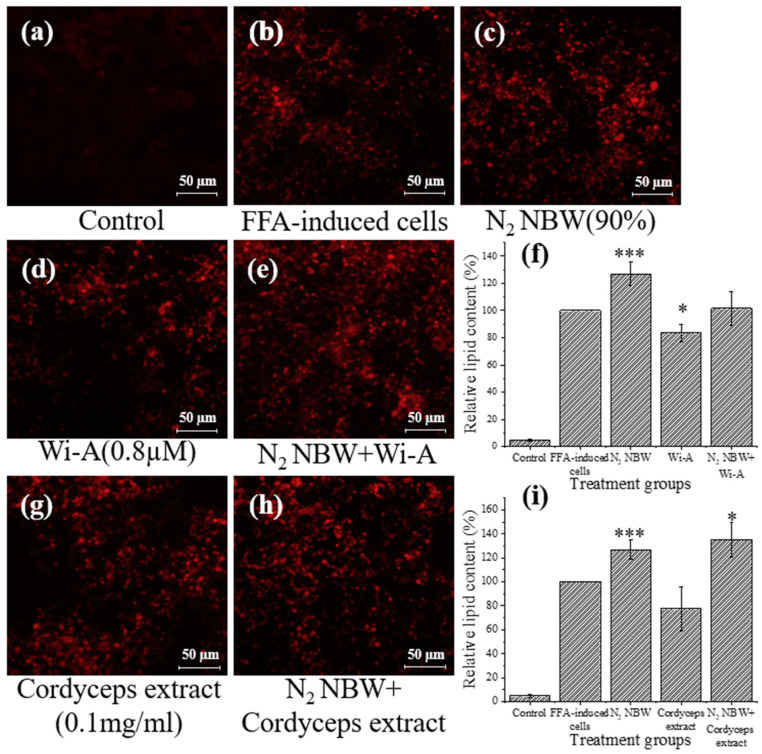
Analysis of lipid content of HepG2 cells treated with nitrogen (N_2_) NBW and with Wi-A and cordyceps extract with and without N_2_ NBW. (**a**) HepG2 cells cultured using DMEM only; (**b**) FFA-exposed HepG2 cells; (**c**) FFA-exposed cells with N_2_ NBW treatment; (**d**) FFA-exposed cells with Wi-A treatment; (**e**) FFA-exposed cells with N_2_ NBW and Wi-A treatment; (**f**) relative lipid content in groups with N_2_ NBW and Wi-A treatment; (**g**) FFA-exposed cells with cordyceps extract treatment; (**h**) FFA-exposed cells with N_2_ NBW and cordyceps extract treatment; (**i**) relative lipid content in groups with N_2_ NBW and cordyceps extract treatment. Relative lipid content was calculated based on red area of FFA-exposed cells (**b**) using ImageJ. Significant differences in lipid content at each condition were compared to that of FFA-exposed cells. *, *p* < 0.05; ***, *p* < 0.001.

As shown in Figure 3c, the lipid content of the FFA-exposed cells increased significantly by approximately 26% when treated with nitrogen NBW, while the Wi-A and cordyceps extract treatments reduced lipid accumulation by 16% and 22%, respectively (Figure 3d,g). As a result, the combination of nitrogen NBW with Wi-A and cordyceps extract resulted in increased intracellular lipid content (Figure 3e,h), especially when combined with cordyceps extract, which increased the lipid content by 35% compared to FFA-exposed cells.

### 3.4. Synergistic Effect of Hydrogen NBW with Natural Compounds

**Figure 4:**
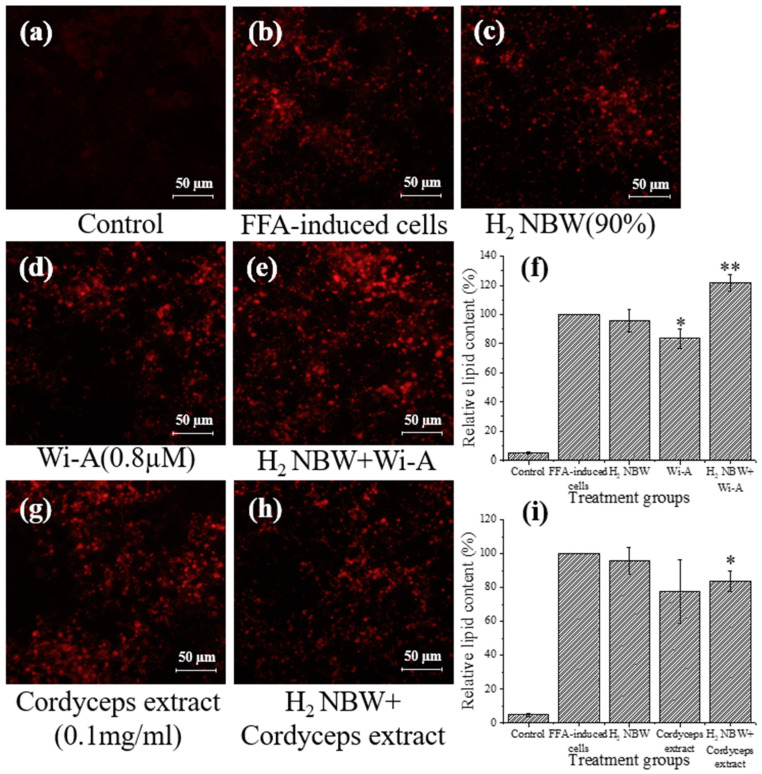
Analysis of lipid content of HepG2 cells treated with hydrogen (H_2_) NBW and with Wi-A and cordyceps extract with and without H_2_ NBW. (**a**) HepG2 cells cultured using DMEM only; (**b**) FFA-exposed HepG2 cells; (**c**) FFA-exposed cells with H_2_ NBW treatment; (**d**) FFA-exposed cells with Wi-A treatment; (**e**) FFA-exposed cells with H_2_ NBW and Wi-A treatment; (**f**) relative lipid content in groups with H_2_ NBW and Wi-A treatment; (**g**) FFA-exposed cells with cordyceps extract treatment; (**h**) FFA-exposed cells with H_2_ NBW and cordyceps extract treatment; (**i**) relative lipid content in groups with H_2_ NBW and cordyceps extract treatment. Relative lipid content was calculated based on red area of FFA-exposed cells (**b**) using ImageJ. Significant differences in lipid content at each condition were compared to that of FFA-exposed cells. *, *p* < 0.05; **, *p* < 0.01.

As shown in Figure 4, hydrogen NBW treatment did not significantly change the lipid content in FFA-exposed cells. However, 38% and 6% increases in lipid content were observed by H_2_ NBW compared to the groups treated with Wi-A and cordyceps extract only (Figure 4d–i).

### 3.5. Synergistic Effect of Oxygen NBW with Natural Compounds

The results of the oxygen NBW treatment differed from those of the nitrogen NBW and hydrogen NBW treatments. Although individual treatment with oxygen NBW slightly increased lipid content compared to the FFA-exposed cells, no significant difference was observed. When oxygen NBW was combined with Wi-A and cordyceps extract, the lipid content decreased by 29% and 33% in FFA-exposed cells. The lipid reduction was slightly (*p* = 0.0843 and *p* = 0.3134, respectively) more pronounced compared to the treatment with each of the two natural compounds only.

## 4. Discussion

NBW is a kind of water that contains large amounts of bubbles with sizes ranging from tens to hundreds of nanometers [22]. The International Organization for Standardization (ISO) certified that all the bubbles with a size below one micron can be named as nanobubbles [23]. NBW has many special physicochemical properties, such as high dissolved oxygen levels (oxygen or air NBW), high stability, and high chemical reaction rates [24], which were verified to enhance plant and microbial growth [16,25]. Nanobubbles can also remain stable in the presence of inorganic salts and organic matter [26]. The high stability of nanobubbles could be owing to many factors, especially the tiny bubble size and the high absolute zeta potential value. It has been reported that nanobubbles in NBW could be stable for more than two weeks when their particle size ranged from 10 to 500 nm [27]. In this study, the particle sizes of nanobubbles in all three NBWs were in the range of 130–200 nm (Figure 1b) and they were stable during the 48-h storage period, which was consistent with the findings of previous studies. Moreover, no significant difference in particle size was observed among the three NBWs during the 48 h.

The zeta potential could be employed to measure the strength of the mutual repulsion or attraction between particles. The higher zeta potential indicates greater mutual repulsion force between the particles, which would reduce the possibility of aggregation, thus increasing stability [28]. Therefore, zeta potential is usually determined to characterize the stability of nanobubbles. It has been reported that the zeta potential of stable NBW can fluctuate from −45 mV to −34 mV and from −20 mV to −17 mV [29]. Moreover, the zeta potential of air NBW produced by different methods could be maintained at −25 mV to −30 mV [30]. In this study, the zeta potential values of all three NBWs remained below −22 mV during the 48 h (Figure 1a). Although the hydrogen NBW showed large fluctuations in the zeta potential value, it still remained below −22 mV. Compared to hydrogen and nitrogen NBWs, oxygen NBW had a higher absolute zeta potential value, indicating that oxygen NBW is the most stable of the three kinds of NBW. Considering the slight alteration in particle sizes and zeta potential values, the physical properties of all three NBWs were relatively stable during the 48-h storage period. Thus, they were suitable for use in subsequent experiments to evaluate their synergistic effects with bioactive substances on lipid reduction.

**Figure 5:**
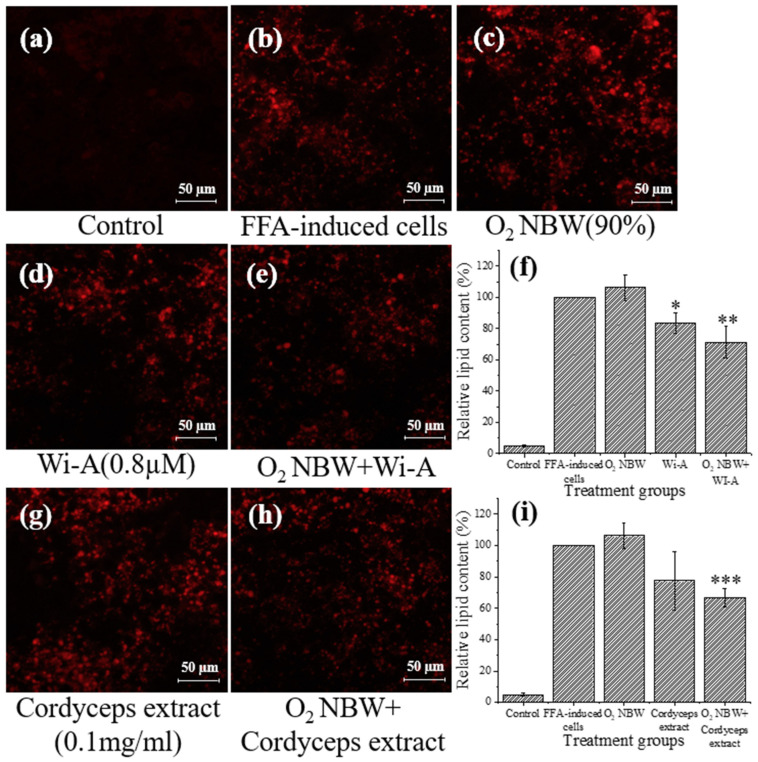
Analysis of lipid content of HepG2 cells treated with oxygen (O_2_) NBW and with Wi-A and cordyceps extract with and without O_2_ NBW. (**a**) HepG2 cells cultured using DMEM only; (**b**) FFA-exposed HepG2 cells; (**c**) FFA-exposed cells with O_2_ NBW treatment; (**d**) FFA-exposed cells with Wi-A treatment; (**e**) FFA-exposed cells with O_2_ NBW and Wi-A treatment; (**f**) relative lipid content in groups with O_2_ NBW and Wi-A treatment; (**g**) FFA-exposed cells with cordyceps extract treatment; (**h**) FFA-exposed cells with O_2_ NBW and cordyceps extract treatment; (**i**) relative lipid content in groups with O_2_ NBW and cordyceps extract treatment. Relative lipid content was calculated based on red area of FFA-exposed cells (**b**) using ImageJ. Significant differences in lipid content at each condition were compared to that of FFA-treated cells. *, *p* < 0.05; **, *p* < 0.01; ***, *p* < 0.001.

Previous studies indicated that both Wi-A and cordyceps extract could be used to reduce lipid accumulation in HepG2 cells. For example, Xiao et al. found that cordycepin polysaccharides could improve the antioxidant capacity and reduce apoptosis of HepG2 cells [31]. Li et al. found that cordyceps could induce cellular autophagy through the activation of the PKA/mTOR pathway to alleviate HepG2 lipid deposition [11]. Shiragannavar et al. verified that Wi-A could improve nonalcoholic fatty liver induced by a high-fat diet [12]. The present study confirmed the results of previous studies. The treatment by Wi-A and cordyceps extract reduced lipid content in FFA-exposed HepG2 cells by 16% and 22%, respectively (Figure 3, Figure 4 and Figure 5).

Hypoxia was found to accelerate fatty liver formation by creating a hypoxic environment and accelerating lipid accumulation in hepatocytes [32,33]. Aron-Wisnewsky et al. found that intermittent hypoxia is a key factor in the development of fatty liver in obese people [34]. Mylonis et al. found that the inhibition of β-oxidation by downregulating peroxisome proliferator-activated receptor gamma coactivator 1-alpha (PGC-1α) and carnitine palmitoyltransferase I (CPT1) further supports lipid accumulation under hypoxic conditions [35]. In the present study, the three types of NBW did not reduce lipid content in FFA-exposed HepG2 cells. Conversely, the lipid content in FFA-exposed HepG2 cells increased under the intervention of nitrogen NBW and hydrogen NBW (Figure 3c and Figure 4c). The low DO values of these two NBWs might be the key reason for the increased lipid content. The DO of deionized water is about 8.98 mg/L at room temperature (25 °C) and atmospheric pressure (1013.25 hPa). However, the DO values of nitrogen NBW and hydrogen NBW are lower than this value [16]. In addition, it has been confirmed that oxygen NBW has the highest DO among different NBWs [29]. Our results were consistent with these studies. Although the DO in the oxygen NBW tended to decrease (from 20 to 12 mg/L) during the 48-h storage period, it was still higher than the DO in water under atmospheric pressure conditions. One could speculate that the oxygen NBW might continuously supply oxygen to increase the DO in water during the gradual decrease in nanobubble concentration. In contrast, the hydrogen NBW and the nitrogen NBW possess extremely low DO levels (4–8 mg/L during 48 h). Although the DO in these two NBWs increased in the later stage of storage, it was still lower than the DO in water at atmospheric pressure. These results indicated that hydrogen and nitrogen NBW treatment might create a hypoxia environment for cell growth, thus increasing the lipid accumulation in the FFA-exposed HepG2 cells (Figure 3 and Figure 4). On the contrary, oxygen NBW treatment did not significantly change lipid content in FFA-exposed cells (Figure 5). From these results, we speculated that simply improving the hypoxic environment does not reduce lipid accumulation or decrease the rate of lipid accumulation.

A previous study found that NBW could efficiently improve substance delivery [36]. Thus, we further attempted to verify the synergistic effects of NBWs and natural compounds on the reduction in lipid accumulation in FFA-exposed HepG2 cells. The results showed that hydrogen NBW and nitrogen NBW are unable to improve the effects of the two natural components on lipid reduction, which might be due to their low oxygen content. However, oxygen NBW exhibited a synergistic effect with natural compounds on lipid reduction. In contrast, the oxygen NBW might alleviate the hypoxic environment for cell growth. However, the high absolute zeta potential value of oxygen NBW might be associated with the better performance on the delivery of natural substances compared to the hydrogen and nitrogen NBWs [16,36].

This study only confirmed the effect of NBW on lipid accumulation via in vitro cell experiments, which did not consider the changes in NBW properties in the complex environment of the human body. More advanced in vitro models or animal experiments should be considered in future studies. Moreover, the potential side effects of NBW, such as its effects on oxidative stress, should be further studied and clarified.

## 5. Conclusions

In this study, the lipid-reducing effects of NBWs made from hydrogen, nitrogen, and oxygen and their combination with natural compounds were investigated in an NAFLD cell model (FFA-exposed HepG2 cells). The results showed that both Wi-A and cordyceps extract treatments effectively reduced lipid accumulation in FFA-exposed HepG2 cells. However, the three NBWs did not reduce lipid accumulation, especially as hydrogen and nitrogen NBWs even promoted lipid accumulation. In addition, the hydrogen and nitrogen NBWs increased lipid accumulation when combined with Wi-A and cordyceps extract, which might be due to the hypoxic environment they provided for cell growth. In contrast, the oxygen NBW promoted the lipid-reducing effect of the two natural compounds, which might be due to high dissolved oxygen environment and facilitated substances transport. In future, the protein expression of relevant signaling pathways should be investigated and combined with multiple omics techniques such as metabolomics and transcriptomics to verify the mechanisms of action.

## Data Availability

Data produced in this study can be made available upon reasonable request.
